# IMRT for locally advanced anal cancer: clinical experience of the Montpellier Cancer Center

**DOI:** 10.1186/1748-717X-7-45

**Published:** 2012-03-23

**Authors:** Sabine Vieillot, Pascal Fenoglietto, Claire Lemanski, Carmen Llacer Moscardo, Sophie Gourgou, Jean-Bernard Dubois, Norbert Aillères, David Azria

**Affiliations:** 1 Département de Cancérologie Radiothérapie, CRLC Val d'Aurelle-Paul Lamarque, Montpellier, France; 2 Unité de Biostatistiques, CRLC Val d'Aurelle-Paul Lamarque, Montpellier, France

**Keywords:** IMRT, Anal canal cancer, Acute toxicity, Late toxicity

## Abstract

**Purpose:**

To assess outcomes of patients with carcinoma of the anal canal (CAC) treated with intensity-modulated radiation therapy (IMRT).

**Method and materials:**

From August 2007 to January 2011, seventy-two patients suffering from CAC were treated with IMRT. Concurrent chemotherapy was added in case of locally advanced tumors. Radiation course consisted in delivering an initial plan to the PTV1 defined as the primary tumor and the risk area including pelvic and inguinal nodes. Forty-five Gy in daily 1.8 Gy-daily fractions were delivered five days a week. A second plan of 14.4-20 Gy to the primary tumor (PTV2) was administered in 1.8-2 Gy-daily fractions, 5 days a week. We present here the results of dosimetry, toxicities, and clinical outcome of the first 39 patients with a median follow-up of 24 months.

**Results:**

Thirty-one women and eight men were included in the present analysis. Tumors were classified as stages I, II, III and IV in 2, 7, 27 and 2 patients, respectively. Median age was 59 years (range, 38-85). Radiotherapy alone (RT) or combined with chemotherapy (RCT) were delivered in 6 (15%) and 33 (85%) patients, respectively.

Six patients (15%) required a treatment break ≥ 3 days, and median time for treatment break was 8 days (range, 3-14 days). Acute grade 3 gastrointestinal (GI) and genitourinary (GU) toxicities were seen in 10 and 5% of patients, respectively. Grade 4 toxicity was only hematologic and occurred in 12% patients receiving RCT. With a median follow-up of 24 months, no patient experienced any late grade 4 toxicity. The 2-year overall survival rate was 89%, the 2-year local relapse free survival was 77% and the 2-year colostomy-free survival rate was 85%.

**Conclusion:**

IMRT is well tolerated with acceptable treatment interruption allowing dose escalation.

## Introduction

For over four decades, the organ-preserving approach based on radiotherapy combined with chemotherapy (RCT) has been preferred over radical surgery or radiotherapy alone for the treatment of locally advanced anal cancer. This strategy has resulted in equivalent overall survival but higher colostomy-free survival (CFS) rates [1-5]. Nevertheless, potential toxicity of exclusive RCT can increase the incidence of treatment breaks and the overall treatment time, which may negatively influence local outcome [6,7]. Pelvic intensity-modulated radiation therapy (IMRT) has been shown to deliver radiation doses in a more conformational way than conventional therapy, thereby reducing doses given to neighboring organs at risk (OAR). Some dosimetric studies about the use of IMRT in anal canal carcinoma have already been published, and indeed reported significant reduction in the doses delivered to the bowel, bladder and genitalia/perineal skin [8-10]. These dosimetric findings were correlated with lower rates of acute and late gastrointestinal (GI) and genitourinary (GU) morbidity [11-13]. More recently, several studies have shown decreased hematologic toxicity by using bone marrow (BM) sparing IMRT [14-17].

The present article details our single institution experience of the first 39 patients with squamous cell carcinomas of the anal canal treated with IMRT +/- chemotherapy (CT). Our procedures for target volume definition, optimization criteria and field arrangements are described. Also, we present dosimetric parameters and early results in terms of toxicity and **oncologic **outcome.

## Methods and materials

From August 2007 to January 2011, 72 patients with histologically confirmed squamous cell carcinoma of the anal canal were treated using IMRT alone or concurrent CT. All patients were evaluated with a complete history and physical examination. Tumor **classification **was defined according to the American Joint Committee on Cancer AJCC 2002 Guidelines [18]. Computed tomography (CT) scan and/or PET-CT were performed for regional and distant staging, endoscopic ultra sound and/or magnetic resonance imaging for local staging. HIV status and other comorbidities were recorded.

### Simulation and target contouring

Patients underwent CT-based simulation (General Electric Systems, Cleveland, OH) with 2.5 mm thick slices from the mid-dorsal spine to the mid-femur. Patients were simulated in the supine position without any custom immobilization device. Most patients had intravenous contrast administered, and a lead wire placed as anal marker. Target and **organs at risk **were manually contoured using the Advantage Sim Software V7.4.5 on the CT-scan slices according to the ICRU 50 [19]. The Gross Tumor Volume (GTV) consisted in the primary tumor and involved nodes. The Clinical Target Volume 1 (CTV1) included the GTV expanded by a 1-cm margin, the anal canal and draining lymphatic areas (perirectal, internal iliac, external iliac, obturator, and inguinal nodes). Presacral nodes were also included for patients with N2-3 and/or T4 tumors. These lymphatic areas were obtained by a 1-cm expansion around the contrast enhanced vessels, but excluding bone or muscles in concordance with recent published guidelines [20]. Finally, a 1-cm margin was added to the CTV to create the Planning Target Volume 1 (PTV1). The boost volume (PTV2) was defined as the pretreatment GTV uniformly expanded by 1.5 cm. The bowel (identified by the small and large intestine segments except the rectum slides within the PTV), rectum, bladder, external genitalia/perineal skin (penis and scrotum in men and vulva in women), iliac crests (from the bony top to the superior part of acetabulum inferiorly) and femoral heads (from the bony top to the lesser trochanter inferiorly) were delineated. Both rectum and bladder were defined as volumes within the respective outer wall contour including contents. For bowel and bladder, a virtual volume excluding the PTV was created (bowel-PTV and bladder-PTV) to avoid hot spots in this volume part, which is outside the PTV. In addition, this method helped us to improve planning target coverage.

### IMRT planning field arrangements, dose prescription and optimization criteria

IMRT plans were generated using commercial inverse planning software (Eclipse, Helios, version 7.2.34, Varian, Palo Alto, CA). Beam geometry consisted of seven coplanar fields for the whole pelvis (phase 1) with the following gantry angles: 0°, 45°, 110°, 180°, 250°, 315°. A 5-field technique was used for the IMRT boost (45°, 110°, 180°, 250°, and 315°). Patients were treated with an 18-MV linear accelerator with a millennium dynamic multileaf collimator (MLC) (21 EX, Varian, Palo Alto, CA). Rationale for this treatment procedure was based on a former study conducted at our institution [10]. The prescribed dose for the initial IMRT plan was 45 Gy to the PTV1 (daily 1.8-Gy fractions, 5 days a week), immediately followed by a second IMRT plan delivering 14.4 Gy (daily 1.8-Gy fractions, 5 days a week) or 20 Gy to the PTV2 (daily 2-Gy fractions, 5 days a week), depending on the physician's choice. The same isocenter defined during the virtual simulation was used throughout treatment.

The OAR optimization constraints were iteratively adjusted while PTV constraints remained fixed until a clinically acceptable treatment plan was obtained. Typical input dose-volume starting constraints for PTV and OAR are described in Tables 1 and 2. During optimization, modification of these values was allowed to minimize the dose received by OAR with an optimal PTV coverage. Each constraint point presented also an associated priority factor defining the relative importance of the given constraint. Treatment plans were considered acceptable with 95% of the PTV receiving ≥ 95% of the prescribed dose, and less than 1% of the PTV receiving more than 109% of the prescribed dose (D1% < 109%). Dose volume histograms (DVH) were calculated for the IMRT plan up to 45 Gy as well as for the total treatment plan to 59.4-65 Gy for the following volumes: PTVs, bowel, bladder, iliac crests, femoral heads and genitalia. The PTV values D98% and D2% (dose received by 98% and 2% of the PTV) were considered to be the maximum and minimum doses.

**Table 1:** Dose-volume starting constraints for PTV

Organ	Volume (%)	Dose (Gy)
PTV1	0	< 49
	100	59
PTV2	0	59
	100	61
PTV1-PTV2	0	56
	100	-

**Table 2:** Dose-volume starting constraints for OAR

Organ	Threshold dose (Gy)	Volume above starting limit (%)*
Bladder	30	80
	40	40
	0	59
Bladder-PTV	40	30
	0	45
Bowel	30	40
	40	30
	0	50
Bowel-PTV	40	20
	0	45
Genitalia/perineum	30	35-45
	40	
	0	48
Iliac bone marrow	10	35-45
	20	25-30
	0	50
Femoral heads	45	5
	0	55

* objective minimize

### Quality assurance

After treatment validation, a former plan was created in the system, copying all the beams included in the treatment plan on a dedicated phantom previously scanned at our institution. All the geometry parameters could be changed but the number of monitor units (MU) and the MLC sequence were exactly the same as in the patient plan. A specific excel sheet was created to collect information of the verification plan. All the plans computed for the treatment were checked before the first day of irradiation. Point measurements and 2D map studies were used. For each patient, a verification plan of each field (with the gantry, table and collimator rotations set to 0°) in an acrylic phantom was generated (PTW, Freiburg, Germany), and values to specific points were taken. These points were not chosen in a high dose or low gradient area but were fixed by phantom geometry. The axis dose in phantom at depth of 6 cm was measured under the accelerator using an ionization chamber with a nominal sensitive volume of 0.125 cc (PTW 31010). Dose measurements, field-by-field, in the isocenter with the arm at 0° in IC showed a difference with the calculation of 2.07 ± 2.83% for 109 fields. The measurements performed with the sum of the beams at the isocenter showed a difference of 2.31 ± 0.56% for the 17 first patients.

To verify relative and/or absolute distribution on 2 dimensions, we selected dosimetric films. A film was placed for each field at 5 cm depth in the phantom and perpendicular to the irradiation. A calibration curve was performed every time we controlled a plan for a patient. Films were developed in an automatic machine and digitized with a Vidar VXR-12 digitizer (Vidar Systems Corporation, Herndon, VA, USA). The spatial resolution used to digitize the film was 75 dpi which corresponded to 2.95 pixels/mm. A non commercial software (Doselab) was used to analyze films by profile and isodose comparison. After 2003, the results were validated using the gamma index. All plans were accepted with 95% of the points with a gamma index > 1 for 3% and 3 mm criteria. Front and lateral electronic portal images were performed daily over the first three treatment fractions, and then weekly for the remaining part of the treatment course. Bony structures in the portal images were matched to the bony anatomy in the digitally reconstructed radiographs.

### Chemotherapy

Concurrent chemotherapy was given to patients with tumors more than 40 mm in size and/or nodal involvement, and when the Karnofsky performance status was higher than 70. It consisted of fluorouracil (800-1000 mg/m^2^/day) administered as a continuous infusion for 96 h (Day 1 = Day 29) combined with either mitomycin C (10-12 mg/m^2^) bolus injection or cisplatin (75-80 mg/m^2^) at the discretion of the physician, both given on Day 1.

### Clinical outcome assessment

Patients were monitored weekly during RT for acute toxicity. After the end of treatment, patients were seen every 3 months the first year, and every 6 months thereafter. Late effects were defined as any side effect that occurred 3 months or later after the end of radiotherapy. Adverse events were scored according to the Common Toxicity Criteria for Adverse Events scale v3.0. Overall survival (OS), disease-free survival (DFS) and CFS were calculated using the Kaplan-Meier method.

## Results

### Patient and tumor characteristics

Of 72 patients with anal cancer treated curatively with IMRT until January 2011 at our institution, the present study included the first thirty-nine patients who benefited from IMRT for the entire duration of treatment. For other patients, radiation boost could be delivered using brachytherapy, external RT or electron therapy.

Patient and tumor characteristics are summarized in Table 3. Median age was 59 years (range, 38-85 years), and most of patients had an ECOG performance status of 0-1. Three patients were known to be HIV-positive or suffering from AIDS. One patient had been treated for a Hodgkin's lymphoma the year before the diagnosis of anal cancer, and had already received 6 cycles of chemotherapy. Three patients had undergone surgical resection before RT treatment, one of whom had presented with positive margins **(initially presented as haemorrhoids)**. Thirty-three patients (85%) were treated by concurrent RCT while 6 patients (15%) received RT alone. The median number of treatment days was 50 (range, 21- 69 days).

**Table 3:** Patient and tumour characteristics

Variable	No. of patients		%
Total No. of patients	39		100
Age, years			
Median		59	
Range		38-85	
Sex			
Male	8		21
Female	31		79
HIV status			
positive	3		8
negative	36		92
T stage			
Tx	1		3
T1	2		5
T2	11		28
T3	18		46
T4	7		18
N stage			
N0	13		46
N1	16		32
N2	10		22
N3	0		0
M stage			
M0	37		95
M1	2		5
Stage			
I	2		5
II	7		20
IIIA	27		70
IV	2		5
Chemotherapy			
No.	33		85
FU/MMC	19		46
FU/cisplatin	11		26
RT dose, Gy			
PTV1 median		45	
range		40-45	
PTV2 median		63	
range		40-65	
RT break ≥ 3 days			
no	31		80
yes	8		20
RT duration (d)			
median		50	
range		(21-69)	

### Radiation delivery and dosimetric parameters

Median radiation doses to the pelvis and to the primary volume were 45 Gy (range, 40-45 Gy) and 63 Gy (range, 40-65 Gy), respectively. OAR optimization constraints were difficult to meet (Table 1) due to the overlap between PTV and bowel. We achieved adequate target coverage of PTV2 with a D2% of 104.4 ± 8%, and a D98% of 98.2 ± 8%. Results of DVH analysis for OAR are reported in Table 4. For bowel, median values of V40 (volume receiving more than 40 Gy) and V30 were 47% and 76%, respectively. The V40 and V30 values were 51% and 84% for bladder, 1.6% and 45% for genitalia/perineum, respectively. For iliac crests, median V10 and V20 values were 51% and 35%, respectively.

**Table 4:** Dose-Volume Histograms (DVH) for OAR

	Bowel	Bladder	Femoral heads	Genitalia	Iliac crests
V45% (cc)	24 ± 30 (110 ± 118)	37 ± 17	4 ± 6	0 ± 10	-
range	0-188 (2-484)	7.5-75	0-26	0-67	
V40% (cc)	47 ± 45 (198 ± 164)	51 ± 16	-	1.6 ± 14	-
range	6-303 (22-651)	8.5-90		0-75	-
V30% (cc)	76 ± 76 (348 ± 219)	84 ± 11	-	45 ± 25	-
range	25-532 (45-890)	56-100		9.5-97	
V20%	-	-	-	-	35 ± 7
range					24-60
V10%	-	-	-	-	51 ± 11
range					39-86

V*x *%: Volume receiving more than *x *Gy

### Toxicity

Acute and late toxicities are listed in Tables 5 and 6, respectively. One patient died from a heart attack during the second course of chemotherapy, i.e. 4 weeks after the start of treatment. Another patient did not receive the last course of CT and had a 1-week RT break after being hospitalized for a massive pulmonary embolus. Two patients prematurely stopped fluorouracil infusion due to cardiac spasms at day 1 (cycle 1 for patient 1, and cycle 2 for patient 2). Both received cisplatin alone for the remaining treatment. One patient had mitomycin-related liver dysfunction, and was therefore given fluorouracil alone for the second CT course. Only 6 patients (15%) required a RT treatment break ≥ 3 days due to grade 2 genitalia/perineum toxicity (n = 2), grade 3 GI toxicity (n = 2), asthenia (n = 1) and pulmonary embolus (n = 1). Median time for treatment break was 8 days (range, 3-14 days).

**Table 5:** Acute Toxicity

Acute toxicity	Grade 2% (n)	Grade 3% (n)	Grade 4% (n)
Skin	42 (16)	42 (16)	0
Gastrointestinal	37 (14)	10 (4)	0
Genitourinary	10 (4)	5 (2)	0
Hemotologic toxicity*	12 (4)	15 (5)	10 (4)
Neutropenia*	0	15 (5)	6 (2)
Anemia*	24 (8)	6 (2)	0
Thrombocytopenia*	9 (3)	3 (1)	6 (2)

* Patients with concurrent RCT (n = 33)

**Table 6:** Late toxicity

Late toxicity	Grade 2% (n)	Grade 3% (n)	Grade 4% (n)
Skin	3 (1)	0	0
Gastrointestinal	25 (7)	7 (2)	0
Genitourinary	14 (4)	0	0
Bone	3 (1)	0	0
Vaginal	28,5 (6)	5 (1)	0
Hematologic *	0	0	0

* Patients with concurrent radiochemotherapy

All acute grade 4 toxicities were hematologic, and encountered in 4 patients (12%) who received concurrent CT, three of them requiring red blood cell transfusion. Overall, there was no acute grade > 3 non hematologic toxicity and the most common treatment-related adverse events were mild or moderate GI toxicity, and grade 2-3 dermatitis. With respect to late toxicity, clinical data were available for 34 patients (21 women and 13 men). There were no grade 4 late effects, and only 3 patients experienced a grade 3 toxicity, including a single case of vaginal fibrosis and 2 cases of GI toxicity (1 anal stenosis and 1 sphincter dysfunction).

### Clinical outcome

Of the 38 patients who completed the full course of RT treatment, complete clinical response, assessed by physical examination, was achieved in 37 patients. A single patient achieved a partial response and required subsequently an abdominoperineal resection. Seven patients had local relapse, associated with synchronous distant metastases in two cases, whereas two patients developed distant metastases without local relapse. With a median follow-up of 24 months (range, 3-52 months), a total of 30 patients were alive without any evidence of disease. The OS and DFS rates at 2 years were 89% and 70%, respectively. The 2-year local relapse-free and CFS rates were 77% and 85%, respectively.

## Discussion

Standard pelvic radiotherapy for anal cancer is typically delivered using opposed anteroposterior and posteroanterior (AP/PA) fields. As a result, extensive portions of bowel, bladder and perineum are included within treatment fields, leading to increased incidences of acute toxicity and chronic sequelae. Likewise, large volumes of bone marrow are encompassed with such field arrangements, which can potentially induce hematologic count depression. While several studies have demonstrated the dosimetric advantage of IMRT over 3-D conformal radiotherapy for OAR and healthy tissue sparing (8-10), only few clinical trials have reported toxicity results and carcinologic outcome of anal cancer patients treated with IMRT [11-13,21]. However, the latter are encouraging and confirm that IMRT has the potential of minimizing acute and late adverse events without compromising target coverage or treatment planning homogeneity. In our study, we observed low rates of GI toxicity (10% of grade 3, and no grade 4), as previously suggested by Pepek et al. and Salama et al. [12,13]. In comparison, Ajani et al. reported nearly 35% of grade 3-4 GI side effects without the use of IMRT [22]. We did not find any relationship between the toxicity grade and the type of CT regimen used. Regarding dosimetric parameters, it appeared that the bowel volumes receiving 30/40 Gy were associated with the occurrence of acute GI grade 2-3 toxicity, but due to the low proportion of events, accurate identification of volume factors influencing toxicity was not possible. Nevertheless, we reported a bowel V30 of about 350 cc (range, 45-890 cc) when Devisetty et al. [21] found a significant correlation between dosimetric parameters and acute GI toxicity for a V30 > 450 cc (33% GI toxicity).

Because daily treatment with full bladder is not much reproducible, even if the bowel is moved away outside the treatment field, most of our patients had an empty bladder during treatment. For a mean bladder volume of 170 cc, we obtained a V40 of 51% (range, 9-90%) and a V30 of 84% (range, 56-100%). Regarding the risk of hematologic toxicity, this is the first study, to our knowledge, using bone marrow sparing-IMRT for the treatment of anal canal. We used V10 and V20 BM constraints which were shown to be predictors of acute hematologic toxicity [16-23], but we considered these constraints only for the iliac crests (22% of active BM) and not for lumbosacral or low pelvic bone marrow [24]. Achieving lumbosacral BM-sparing seems to be very difficult owing to the proximity to the PTV, particularly when presacral region is included. Also, we did not consider specific constraints on sacral BM to optimize the coverage of the PTV and DVH results for bowel and bladder.

Comparing hematologic tolerance, we noted lower rates of grade 3 (15%) and grade 4 (10%) adverse events than those reported from clinical trials investigating the treatment of anal cancer patients without BM-sparing IMRT [11-13]. Yet, we treated with higher radiation doses (median of 61.6 Gy) as compared to those provided by Salama (51.5 Gy) and Milano (54 Gy). Actually, longer follow-up would be warranted to determine if the low level of toxicity observed in our patients will be associated with improvement in long-term functional and oncologic outcomes.

Clinical implementation of IMRT **in monocentric series seems to improve **therapeutic outcome of patients with anal canal carcinoma, especially thanks to better target coverage, higher doses delivered in selected volumes, while more effectively OAR sparing than with 3D conformal radiotherapy. However, a potential drawback of IMRT is that it requires a long treatment delivery time with an average of 14 min. for 7 split fields and 5 min. for the second plan with 5 non split fields, involving large number of MU and greater integral body dose. One of the major advantages of Volumetric Modulated Arc Therapy (VMAT) is that it offers the possibility of identically covering PTV and sparing surrounding tissues with reduced treatment time and lower number of MUs than with IMRT [25].

## Conclusion

The use of IMRT for the treatment of anal canal cancer is associated with low acute and late toxicities allowing dose escalation without treatment break. The downsides of IMRT include long treatment delivery time and high number of MUs that the novel VMAT technique may improve.

## Competing interests

The authors declare that they have no competing interests.

## Authors' contributions

SV, CLM, PF, and DA conceived the study and drafted the manuscript. SV and PF collected data. NA, CLM, JBD, CL participated in coordination and helped to draft the manuscript. SG performed the statistical analyses. DA provided mentorship and edited the manuscript. All authors have read and approved the final manuscript.
